# Supplementary material for: Chemokine expression profile of an innate granuloma
Source: bioRxiv. 2024 Feb 1:2024.01.30.577927. Preprint. [Version 1] doi: 10.1101/2024.01.30.577927 (PMC10862903; doi:10.1101/2024.01.30.577927)
Supplement: Supplementary file 1 [file NIHPP2024.01.30.577927v1-supplement-1.pdf]

bioRxiv preprint doi: https://doi.org/10.1101/2024.01.30.577927; this version posted February 1, 2024. The copyright holder for this preprint (which was not certified by peer review) is the author/funder, who has granted bioRxiv a license to display the preprint in perpetuity. It is made available under aCC-BY 4.0 International license.

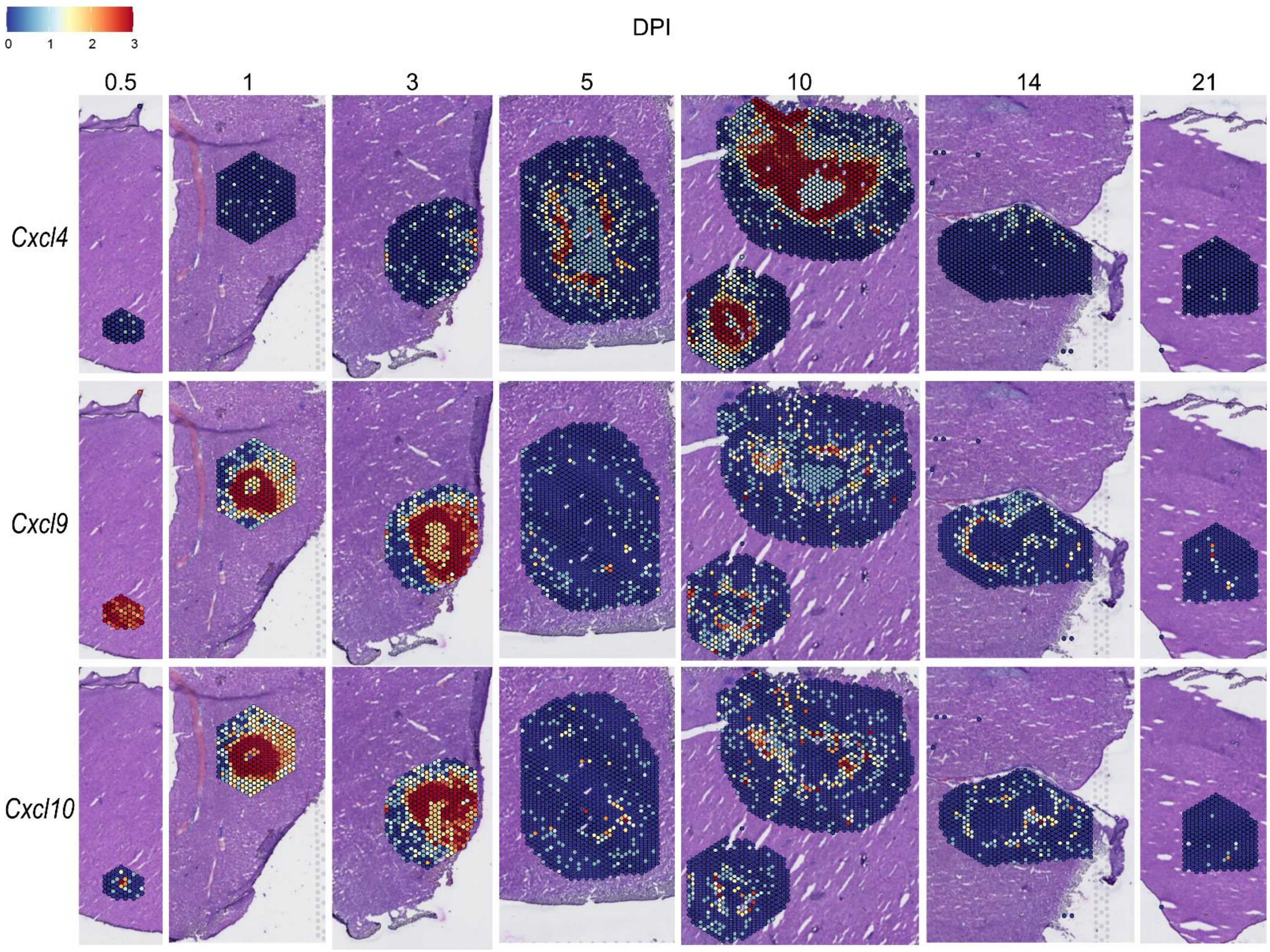

Supplemental Figure 1. Spatial expression of CXCR3 ligands. SpatialFeaturePlots displaying gene expression data of CXCR3 ligands (i.e. *Cxcl4*, *Cxcl9*, and *Cxcl10*) at various days post-infection (DPI). Scale set at 0 – 3.0 expression.

bioRxiv preprint doi: https://doi.org/10.1101/2024.01.30.577927; this version posted February 1, 2024. The copyright holder for this preprint (which was not certified by peer review) is the author/funder, who has granted bioRxiv a license to display the preprint in perpetuity. It is made available under aCC-BY 4.0 International license.

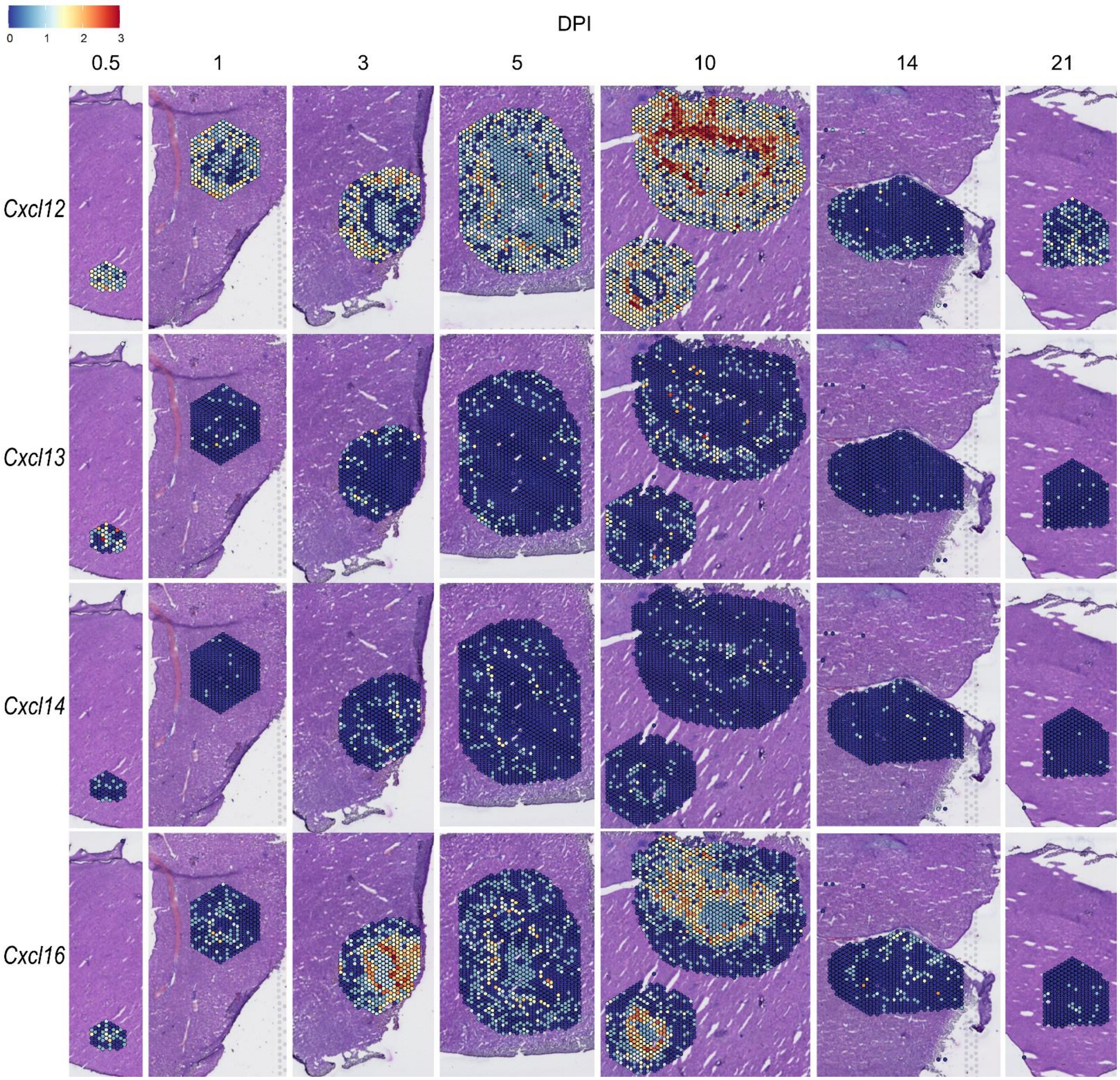

Supplemental Figure 2. Spatial expression of *Cxcl12*, *Cxcl13*, *Cxcl14,* and *Cxcl16*. SpatialFeaturePlots displaying gene expression data of selected *Cxcl* family members at various days post-infection (DPI). Scale set at 0 – 3.0 expression.

bioRxiv preprint doi: https://doi.org/10.1101/2024.01.30.577927; this version posted February 1, 2024. The copyright holder for this preprint (which was not certified by peer review) is the author/funder, who has granted bioRxiv a license to display the preprint in perpetuity. It is made available under aCC-BY 4.0 International license.

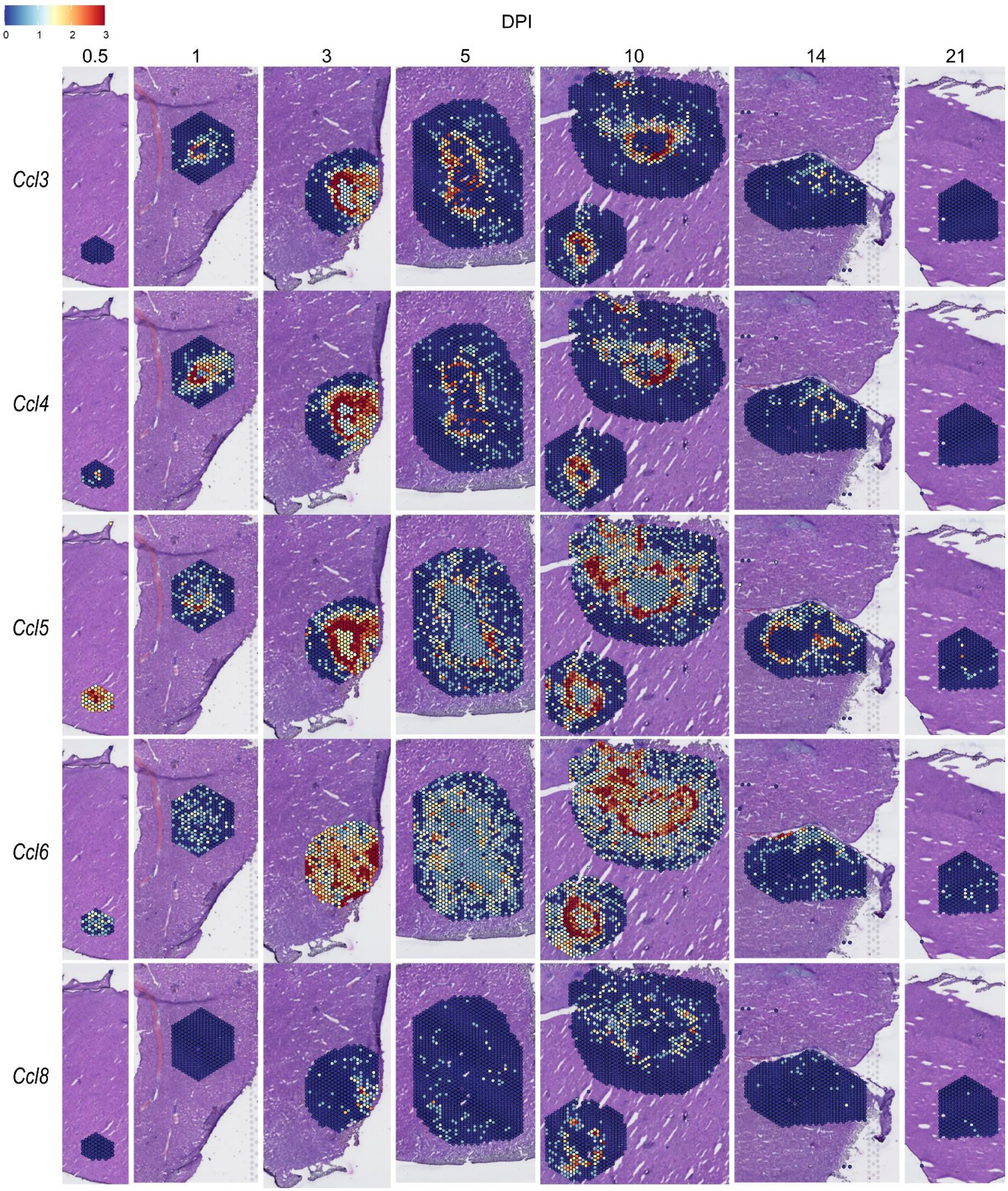

Supplemental Figure 3. Spatial expression of *Ccl3, Ccl4, Ccl5, Ccl6,* and *Ccl8*. SpatialFeaturePlots displaying gene expression data of selected *Ccl* family members at various days post-infection (DPI). Scale set at 0 – 3.0 expression.

bioRxiv preprint doi: https://doi.org/10.1101/2024.01.30.577927; this version posted February 1, 2024. The copyright holder for this preprint (which was not certified by peer review) is the author/funder, who has granted bioRxiv a license to display the preprint in perpetuity. It is made available under aCC-BY 4.0 International license.

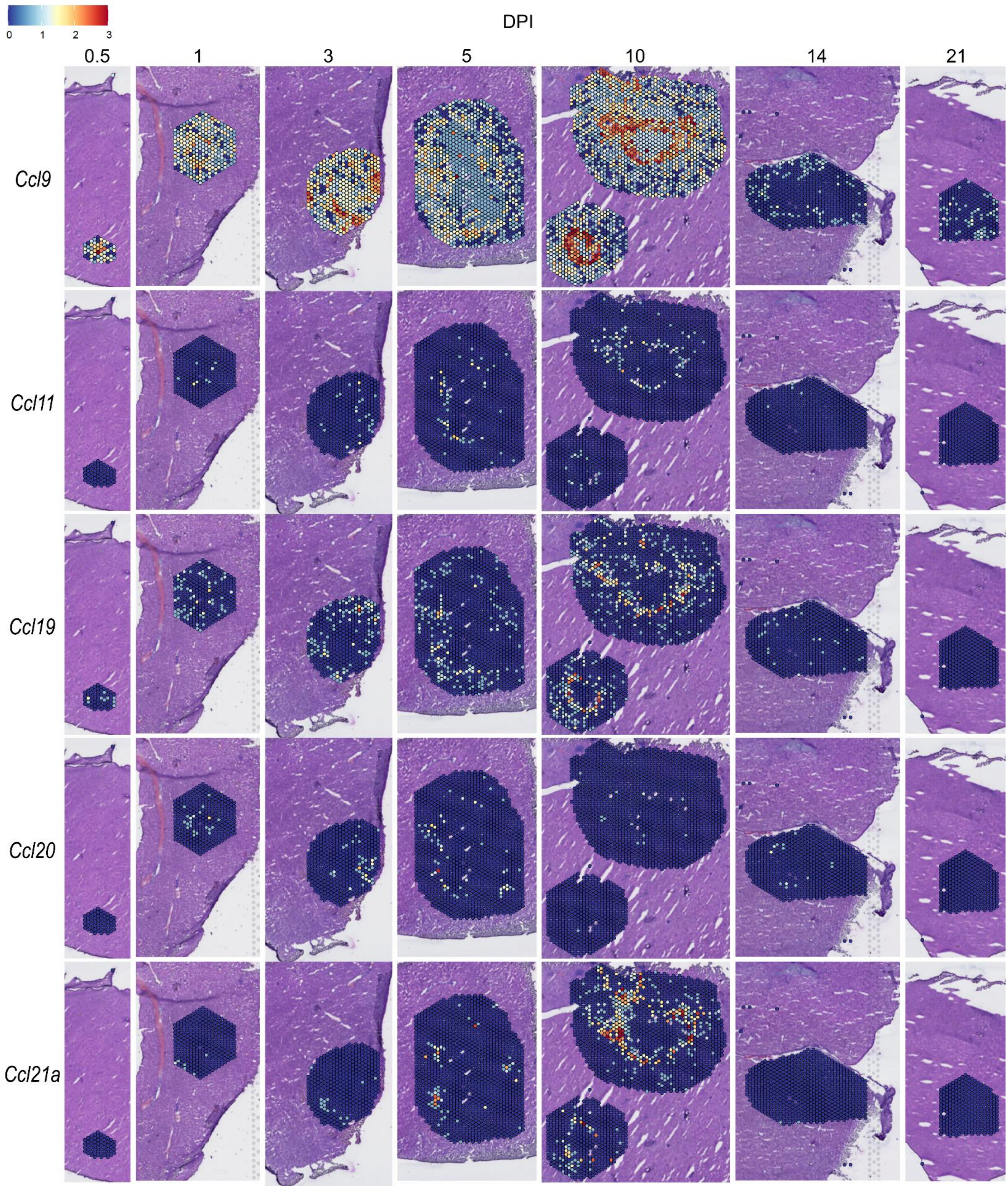

Supplemental Figure 4. Spatial expression of *Ccl9, Ccl11, Ccl19, Ccl20,* and *Ccl21a.* SpatialFeaturePlots displaying gene expression data of selected *Ccl* family members at various days post-infection (DPI). Scale set at 0 – 3.0 expression.

bioRxiv preprint doi: https://doi.org/10.1101/2024.01.30.577927; this version posted February 1, 2024. The copyright holder for this preprint (which was not certified by peer review) is the author/funder, who has granted bioRxiv a license to display the preprint in perpetuity. It is made available under aCC-BY 4.0 International license.

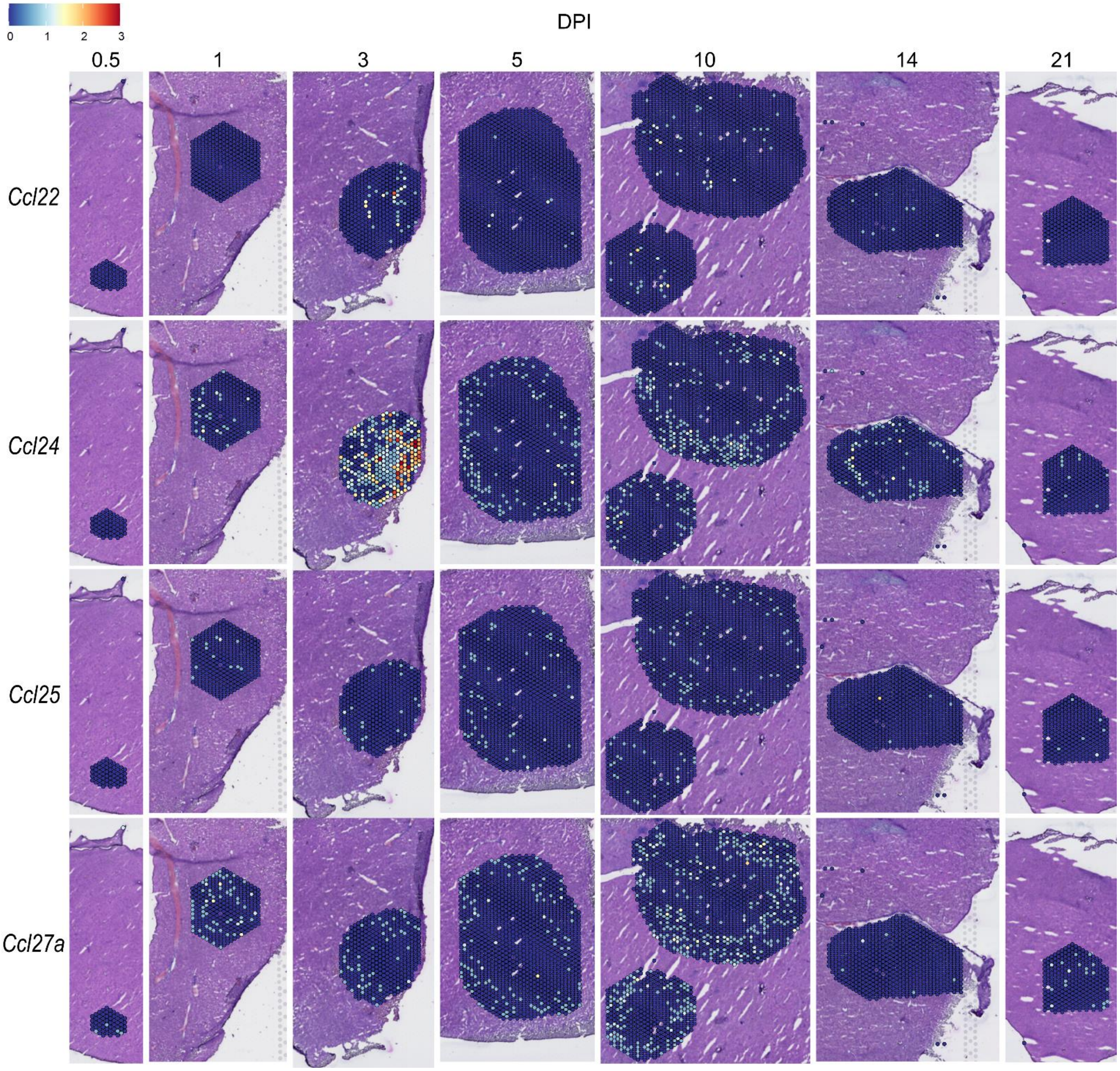

Supplemental Figure 5. Spatial expression of *Ccl22, Ccl24, Ccl25,* and *Ccl27a*. SpatialFeaturePlots displaying gene expression data of selected *Ccl* family members at various days post-infection (DPI). Scale set at 0 – 3.0 expression.

bioRxiv preprint doi: https://doi.org/10.1101/2024.01.30.577927; this version posted February 1, 2024. The copyright holder for this preprint (which was not certified by peer review) is the author/funder, who has granted bioRxiv a license to display the preprint in perpetuity. It is made available under aCC-BY 4.0 International license.

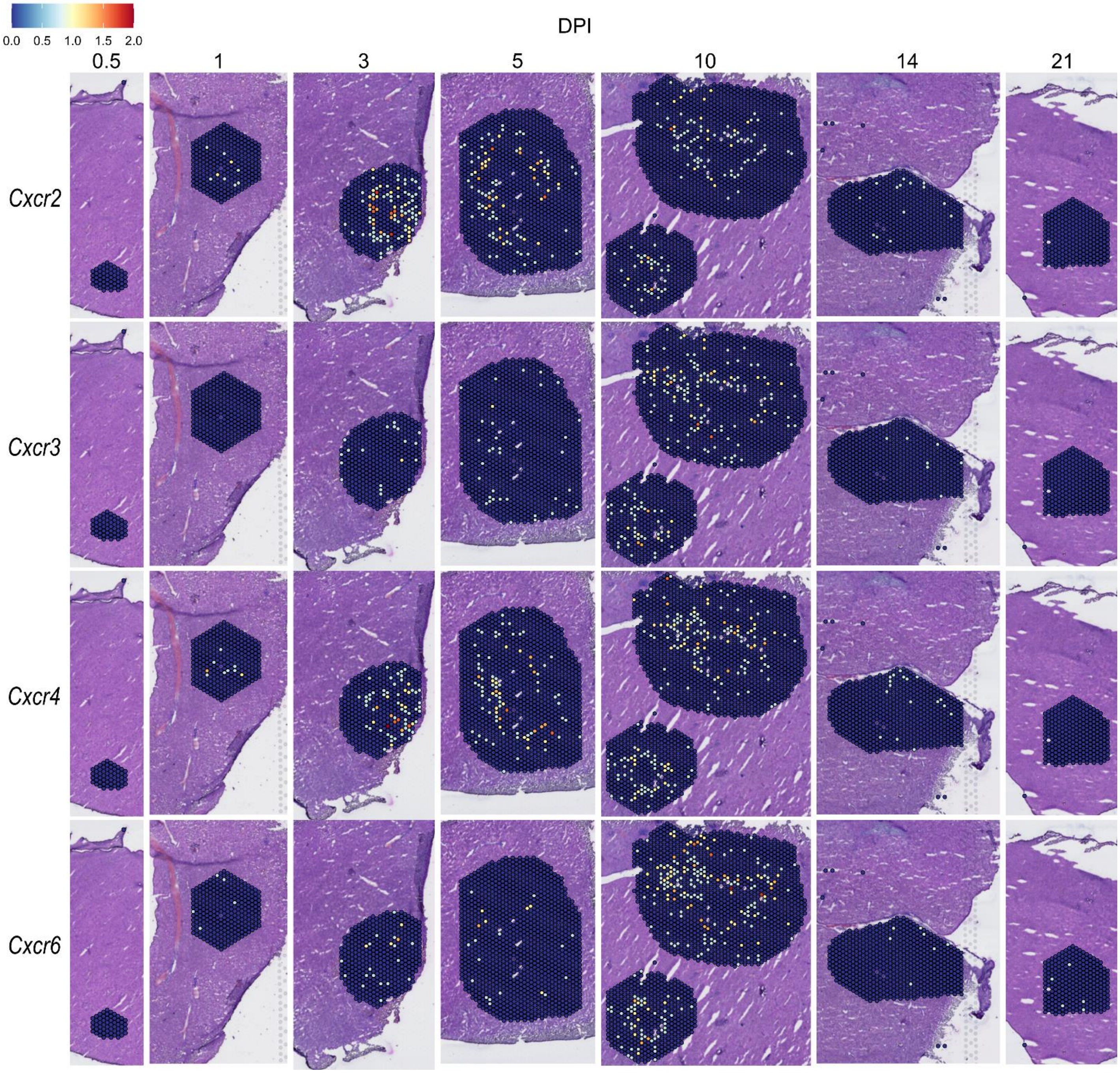

Supplemental Figure 6. Spatial expression of *Cxcr* family members. SpatialFeaturePlots displaying gene expression data of selected *Cxcr* family members at various days post-infection (DPI). Scale set at 0 – 2.0 expression.

bioRxiv preprint doi: https://doi.org/10.1101/2024.01.30.577927; this version posted February 1, 2024. The copyright holder for this preprint (which was not certified by peer review) is the author/funder, who has granted bioRxiv a license to display the preprint in perpetuity. It is made available under aCC-BY 4.0 International license.

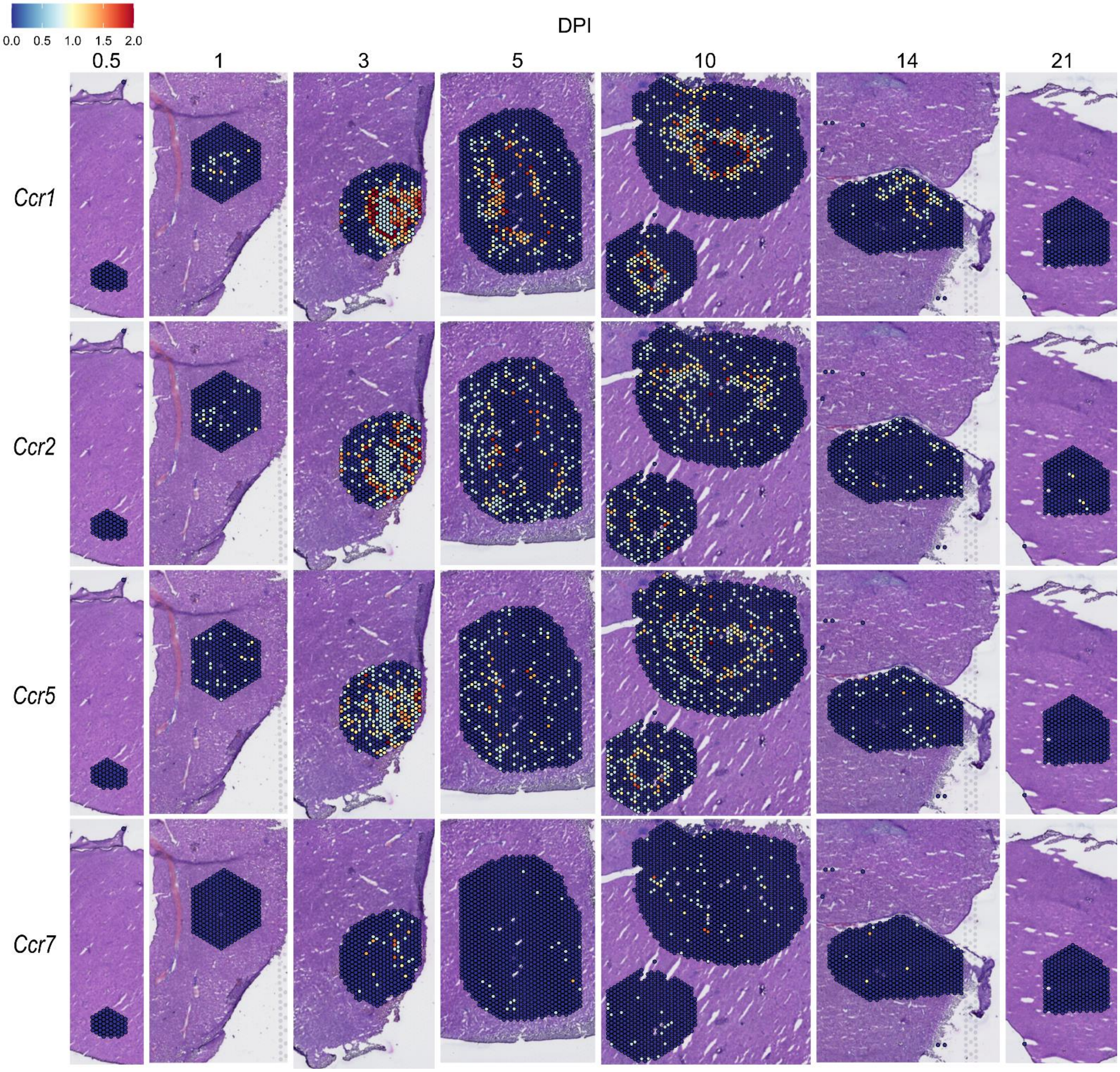

Supplemental Figure 7. Spatial expression of *Ccr* family members. SpatialFeaturePlots displaying gene expression data of selected *Ccr* family members at various days post-infection (DPI). Scale set at 0 – 2.0 expression.

bioRxiv preprint doi: https://doi.org/10.1101/2024.01.30.577927; this version posted February 1, 2024. The copyright holder for this preprint (which was not certified by peer review) is the author/funder, who has granted bioRxiv a license to display the preprint in perpetuity. It is made available under aCC-BY 4.0 International license.

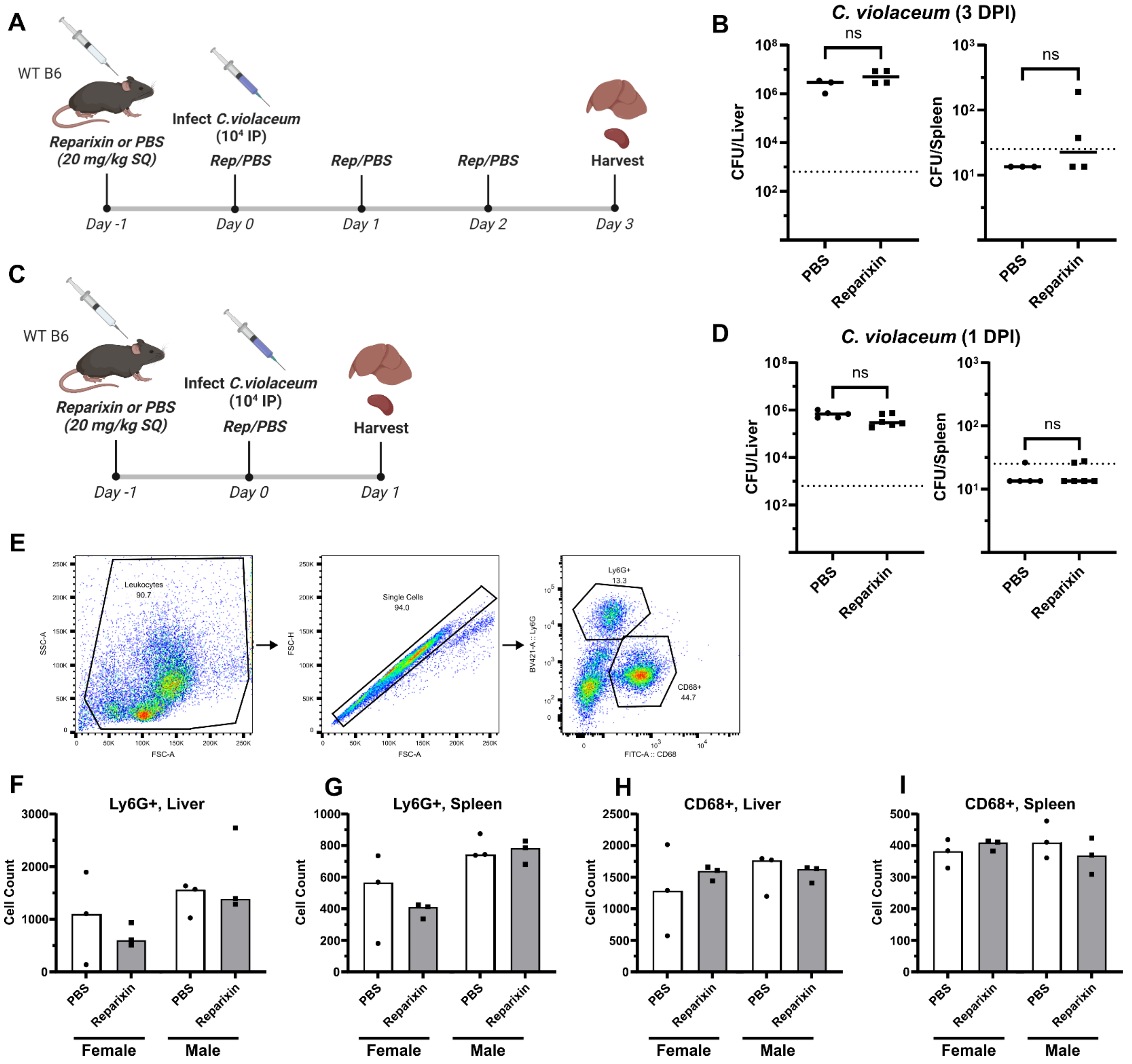

Supplemental Figure 8. Reparixin does not inhibit neutrophil chemotaxis into the liver of infected mice. (**A**) Schematic of the experimental procedure. Mice were injected subcutaneously (SQ) with 20 mg/kg of reparixin, or with PBS. The following day, mice were infected intraperitoneally (IP) with 1x10^4 CFU of *C. violaceum* and treated again with reparixin or PBS. Mice were treated daily thereafter until harvesting on day 3 post-infection. (**B**) Bacterial burdens in the liver and spleen of PBS- or reparixin-treated mice at 3 days post-infection (DPI). (**C**) Schematic of the experimental procedure as in A, except mice were harvested on day 1 post-infection. (**D**) Bacterial burdens in the liver and spleen of PBS- or reparixin-treated mice at 1 DPI. (**E**) Gating analysis of neutrophil ($Ly6G^+$) and macrophage ($CD68^+$) numbers via flow cytometry. Neutrophil numbers in the (**F**) liver and (**G**) spleen. Macrophage numbers in the (**H**) liver and (**I**) spleen. Each dot represents one mouse, with 10,000 events collected per sample. Line at median. (B and D) Dotted line, limit of detection. Solid line, median. Mann-Whitney (abnormally distributed data) for all except liver CFU at 1 DPI, which was analyzed using a two-tailed t test (normally distributed data). Not significant (ns). (B) Liver, p=0.6286; spleen, p=0.4286. (D) Liver, p=0.0641; spleen, p=0.8485. (B) One experiment. (D) Two experiments combined. (F-I) Two experiments combined.

bioRxiv preprint doi: https://doi.org/10.1101/2024.01.30.577927; this version posted February 1, 2024. The copyright holder for this preprint (which was not certified by peer review) is the author/funder, who has granted bioRxiv a license to display the preprint in perpetuity. It is made available under aCC-BY 4.0 International license.

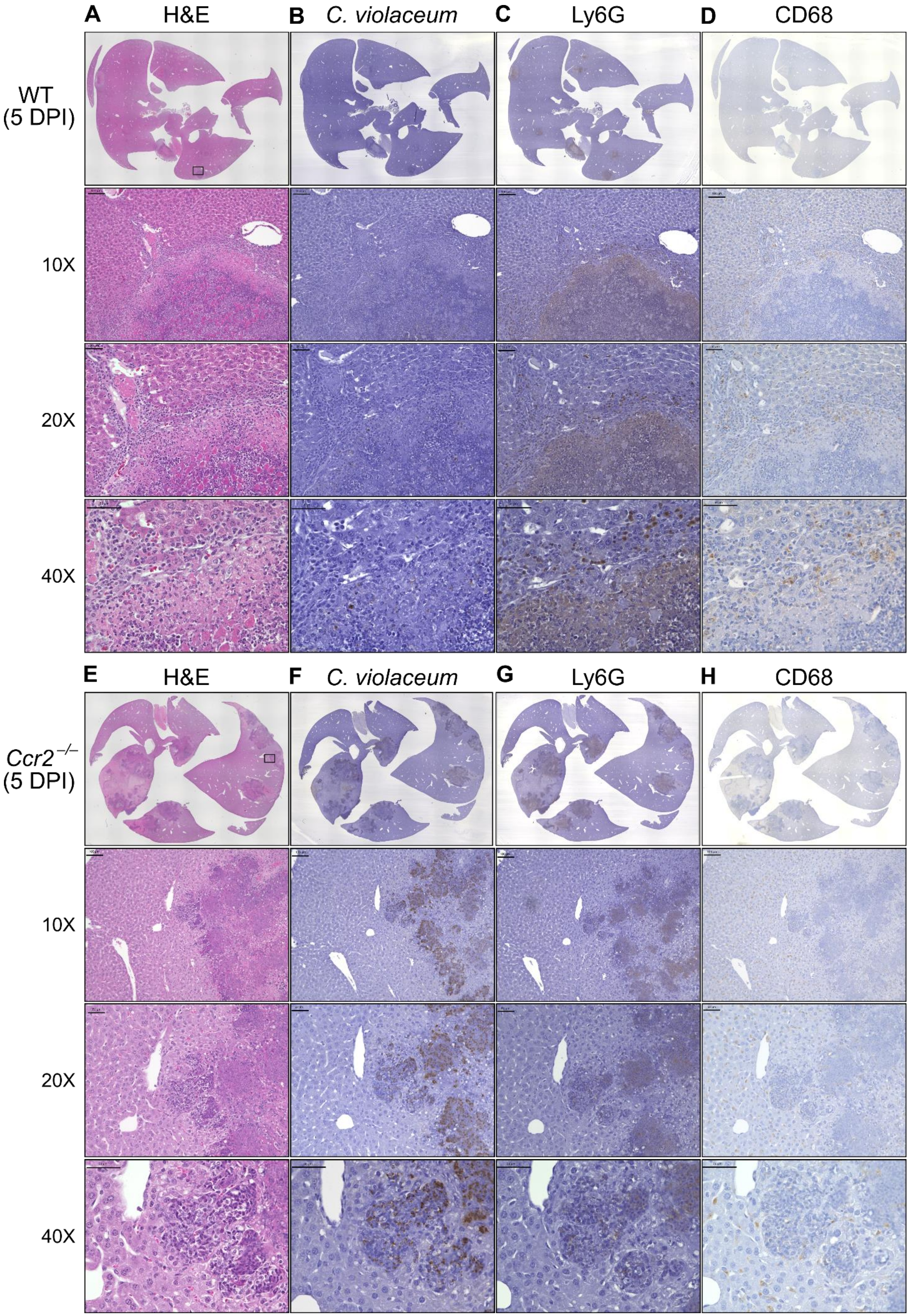

Supplemental Figure 9. Loss of CCR2-dependent monocyte trafficking results in abnormal granuloma architecture and failure of bacterial containment. WT and *Ccr2*$^{-/-}$ mice were infected intraperitoneally (IP) with 1x10^{4}

bioRxiv preprint doi: https://doi.org/10.1101/2024.01.30.577927; this version posted February 1, 2024. The copyright holder for this preprint (which was not certified by peer review) is the author/funder, who has granted bioRxiv a license to display the preprint in perpetuity. It is made available under aCC-BY 4.0 International license.

CFU *C. violaceum* and livers harvested 5 days post-infection (DPI). Serial sections of livers stained by hematoxylin and eosin (H&E) or various IHC markers for (**A-D**) WT female and (**E-H**) *Ccr2*$^{-/-}$ female. For 10X, scale bar is 100 μm. For 20X and 40X, scale bar is 50 μm.

bioRxiv preprint doi: https://doi.org/10.1101/2024.01.30.577927; this version posted February 1, 2024. The copyright holder for this preprint (which was not certified by peer review) is the author/funder, who has granted bioRxiv a license to display the preprint in perpetuity. It is made available under aCC-BY 4.0 International license.

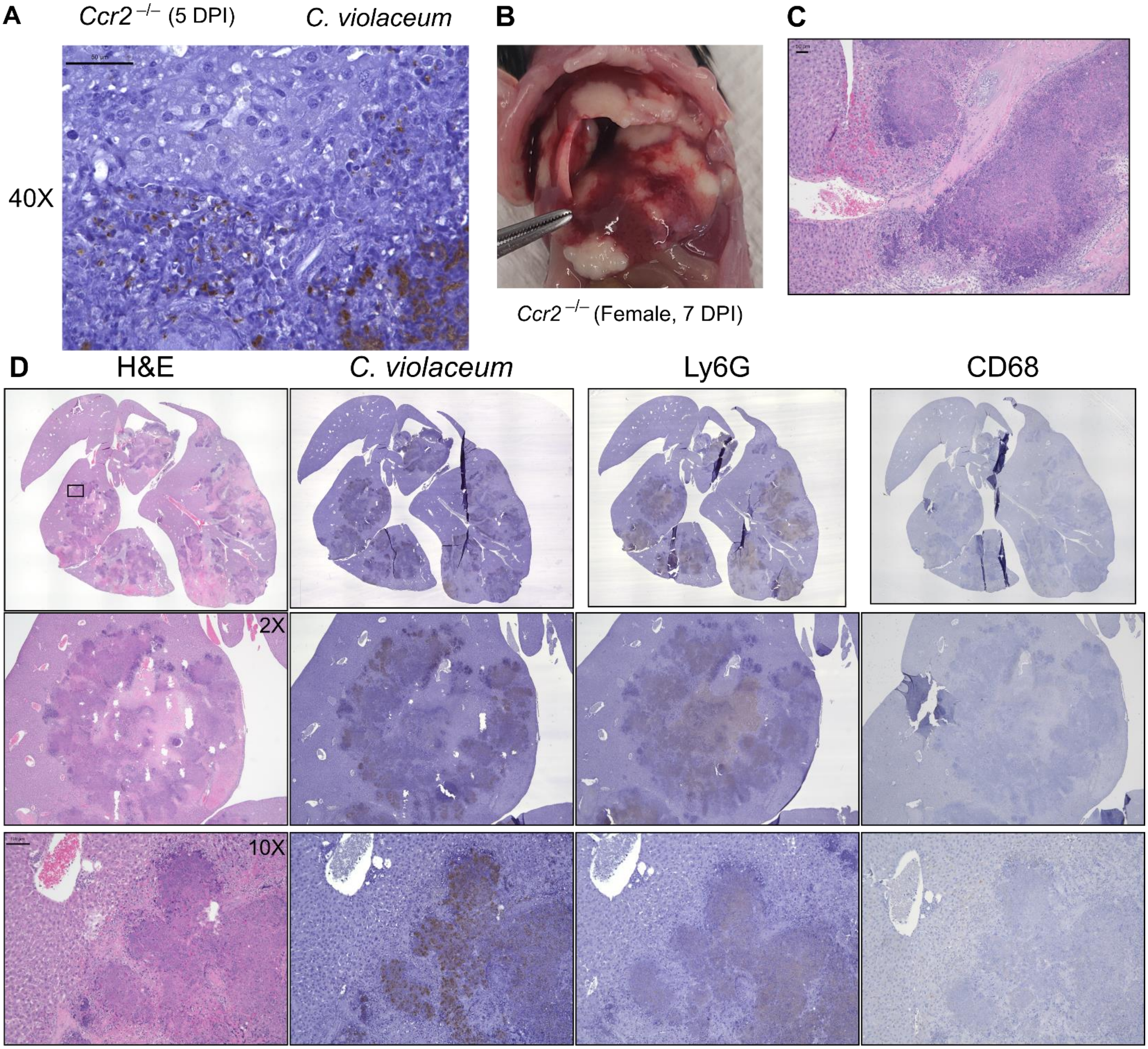

Supplemental Figure 10. *Ccr2*$^{-/-}$ mice have increased necrosis and clotting. *Ccr2*$^{-/-}$ mice were infected intraperitoneally (IP) with 1x10^4 CFU *C. violaceum*. (**A**) Liver section from *Ccr2*$^{-/-}$ male mouse 5 days post-infection (DPI), stained for *C. violaceum*; zoom showing individual puncta of *C. violaceum*. (**B-D**) A *Ccr2*$^{-/-}$ female mouse from survival curve in Figure 6A that was sacrificed at 7 DPI according to euthanasia criteria. (B) Gross pathology. (C) Liver section stained by hematoxylin and eosin (H&E) showing clotting. (D) Serial sections of liver stained with H&E or various IHC markers. For 10X, scale bar is 100 μm.
